# Usefulness of CT scan as part of an institutional protocol for proactive leakage management after low anterior resection for rectal cancer

**DOI:** 10.1007/s00423-022-02652-z

**Published:** 2022-08-25

**Authors:** K. Talboom, C. P. M. van Helsdingen, S. Abdelrahman, J. P. M. Derikx, P. J. Tanis, R. Hompes

**Affiliations:** 1 Department of Surgery, Amsterdam UMC, Location AMC, Meibergdreef 9, 1105 AZ Amsterdam, The Netherlands; 2 Department of Paediatric Surgery, Emma Childrens Hospital, Amsterdam UMC, University of Amsterdam and Vrije Universiteit, Amsterdam Gastroenterology Endocrinology Metabolism Research Institute, Amsterdam, The Netherlands; 3 Department of Surgery, Amsterdam UMC, Location VUmc, Amsterdam, The Netherlands

**Keywords:** Low anterior resection, Anastomotic leakage, Clinical characteristics, CT imaging

## Abstract

**Purpose:**

Highly selective fecal diversion after low anterior resection (LAR) for rectal cancer requires a strict postoperative protocol for early detection of anastomotic leakage (AL). The purpose of this study was to evaluate C-reactive protein (CRP)–based CT imaging in diagnosis and subsequent management of AL.

**Methods:**

All patients that underwent a CT scan for suspicion of AL after transanal total mesorectal excision for rectal cancer in a university center (2015–2020) were included. Outcome parameters were diagnostic yield of CT and timing of CT and subsequent intervention.

**Results:**

Forty-four out of 125 patients underwent CT (35%) with an overall median interval of 5 h (IQR 3–6) from CRP measurement. The anastomosis was diverted in 7/44 (16%). CT was conclusive or highly suspicious for AL in 23, with confirmed AL in all those patients (yield 52%), and was false-negative in one patient (sensitivity 96%). CT initiated subsequent intervention after median 6 h (IQR 3–25). There was no or minor suspicion of AL on imaging in all 20 patients without definitive diagnosis of AL. After CT imaging on day 2, AL was confirmed in 0/1, and these proportions were 6/6 for day 3, 7/10 for day 4, 2/4 for day 5, and 9/23 beyond day 5.

**Conclusion:**

In the setting of an institutional policy of highly selective fecal diversion and pro-active leakage management, the yield of selective CT imaging using predefined CRP cut-off values was 52% with a sensitivity of 96%, enabling timely and tailored intervention after a median of 6 h from imaging.

**Supplementary Information:**

The online version contains supplementary material available at 10.1007/s00423-022-02652-z.

## Introduction

Anastomotic leakage (AL) after low anterior resection (LAR) for rectal cancer is a severe complication with frequent need for reinterventions and readmissions, and is associated with worse oncological outcome, increased health care costs, and decreased quality of life [1–4]. Conventional treatment of AL consists of fecal diversion and drainage of the abscess and a subsequent period of secondary healing, while dismantling of the anastomosis might be performed in more severe cases. More recently, pro-active approaches have been developed using endoscopic vacuum therapy (EVT) and early closure of the anastomotic defect [5, 6]. Early initiation of EVT appears to be more effective, when the presacral cavity is still pliable and unaffected by chronic inflammation, thereby increasing the chance of eventual restoration of bowel continuity.

Timely detection seems important to limit the consequences of AL, but consensus on diagnostic protocols with clear implications for subsequent management is lacking. Clinical parameters indicative of AL include pelvic pain, nausea, tachycardia, tachypnea, hypotension, and fever [7, 8]. Serum C-reactive protein (CRP) levels can be indicative of infectious complications with discriminative power on days 3 and 4 [9]. Both clinical parameters and CRP can result in false-negative and false-positive findings that hamper their use for proper selection of patients who require subsequent invasive diagnostics (e.g., endoscopy, laparoscopy) or immediate surgical treatment. CT imaging can add diagnostic accuracy, but not all radiological features associated with AL are highly sensitive [10, 11].

Fecal diversion might mask the presence of an AL, which results in delayed diagnosis, thereby losing the window of opportunity for early intervention [12]. This was one of the reasons, besides the associated morbidity and need for reinterventions related to a stoma, to implement a policy of highly selective diversion after LAR at our institute. This policy appeared to be safe and did not lead to more complicated leaks, while having low permanent stoma rates [13]. CT imaging with rectal contrast is one of the corner stones of our institutional protocol for early detection of AL with subsequent tailored intervention.

The primary aim of this study was to evaluate the usefulness of CT imaging within an institutional protocol for early detection of AL in patients after transanal total mesorectal excision (TaTME) for rectal cancer with highly selective fecal diversion. The secondary aims of this study were to analyze the yield of CT depending on time interval from index surgery, CRP values at time of imaging relative to predefined cutoff values, sensitivity of the individual radiological features, and timing of initial postoperative CT imaging and subsequent reinterventions.

## Methods

### Study population

A retrospective cohort study was performed, including all patients that underwent CT imaging for suspicion of AL after TaTME for primary mid or distal rectal cancer, with or without temporary diverting stoma operated between April 2015 and December 2020, in the Amsterdam UMC, location AMC. Exclusion criteria were partial mesorectal excision and surgery for recurrent rectal cancer.

### Surgery and perioperative management

All patients underwent TaTME without routine diverting stoma. A policy of highly selective fecal diversion was introduced in our center in 2014 as previously described [14]. All patients received preoperative mechanical bowel preparation and intravenous preoperative antibiotics. Postoperatively, CRP was routinely measured at day 4 until 2019, and on day 3 since then, related to the design of the IMARI study [15]. If CRP levels were elevated above predefined cutoff values and/or there was a clinical suspicion of AL, patients underwent a CT scan with iv contrast and preferably also water-soluble oral and rectal contrast. Cut-off values for CRP were based on a previous review (CRP > 172 mg/L on day 3, > 124 mg/L on day 4, and > 144 mg/L on day 5) [9]. If AL was suspected or clearly visible on CT, subsequent management consisted of endoscopic assessment of the anastomosis and surgical reintervention whenever indicated (e.g., construction of a diverting ileostomy, abdominal lavage for peritonitis). If endoscopy revealed an abscess cavity, EVT-treatment was initiated by placing a vacuum sponge. When the cavity appeared clean with granulation tissue after a few exchanges, the defect was closed with transanal sutures. Details of this technique were published earlier [6].

### Data collection and outcome parameters

Electronic medical files were used for data collection. Data was collected on baseline characteristics, index operation, serum CRP-levels, postoperative imaging, clinical parameters, postoperative complications, and reinterventions. All radiological characteristics, including timing and individual features were collected from the radiology reports. Clinical parameters potentially associated with AL were collected at time of diagnosis of AL or 24 h prior to diagnosis of AL. The primary outcome was the diagnostic yield of CT. Secondary outcomes included individual radiological features indicative for diagnosing AL, mean CRP levels at the time of CT on different postoperative days, proportion of CT with preceding CRP above predefined cutoff levels on different postoperative days, timing of CT imaging, and timing and type of reinterventions for confirmed AL. Eventually confirmed diagnosis of AL was defined as an anastomotic defect found during endoscopy and/or surgery followed by treatment for AL.

### Statistical analysis

Analyses were performed for the whole group of patients who underwent CT for suspicion of AL, and for the subgroups with or without confirmed AL. The data was analyzed using IBM SPSS statistics, version 26.0, Armonk, NY. Chi-square test was used for categorical and dichotomous variables, presented as absolute numbers with percentages. For continuous variables with a normal distribution, an independent sample *T* test was used, and outcomes were reported as mean with standard deviation. In case of a non-normal distribution, a Mann–Whitney *U* test was used to calculate the median with interquartile range. Sensitivity and specificity rates were calculated for anastomotic leakage, using the outcome of the CT scan as testing modality and confirmation of diagnosis by either endoscopy or surgical intervention. Median time intervals in hours were calculated between index operation and first CRP, index operation and first CT scan, index operation and first reintervention, first CRP and CT imaging, and CT imaging and first reintervention. Two sided *p* values were calculated and considered statistically significant if *p* < 0.05.

## Results

### Study population

**Fig. 1:**
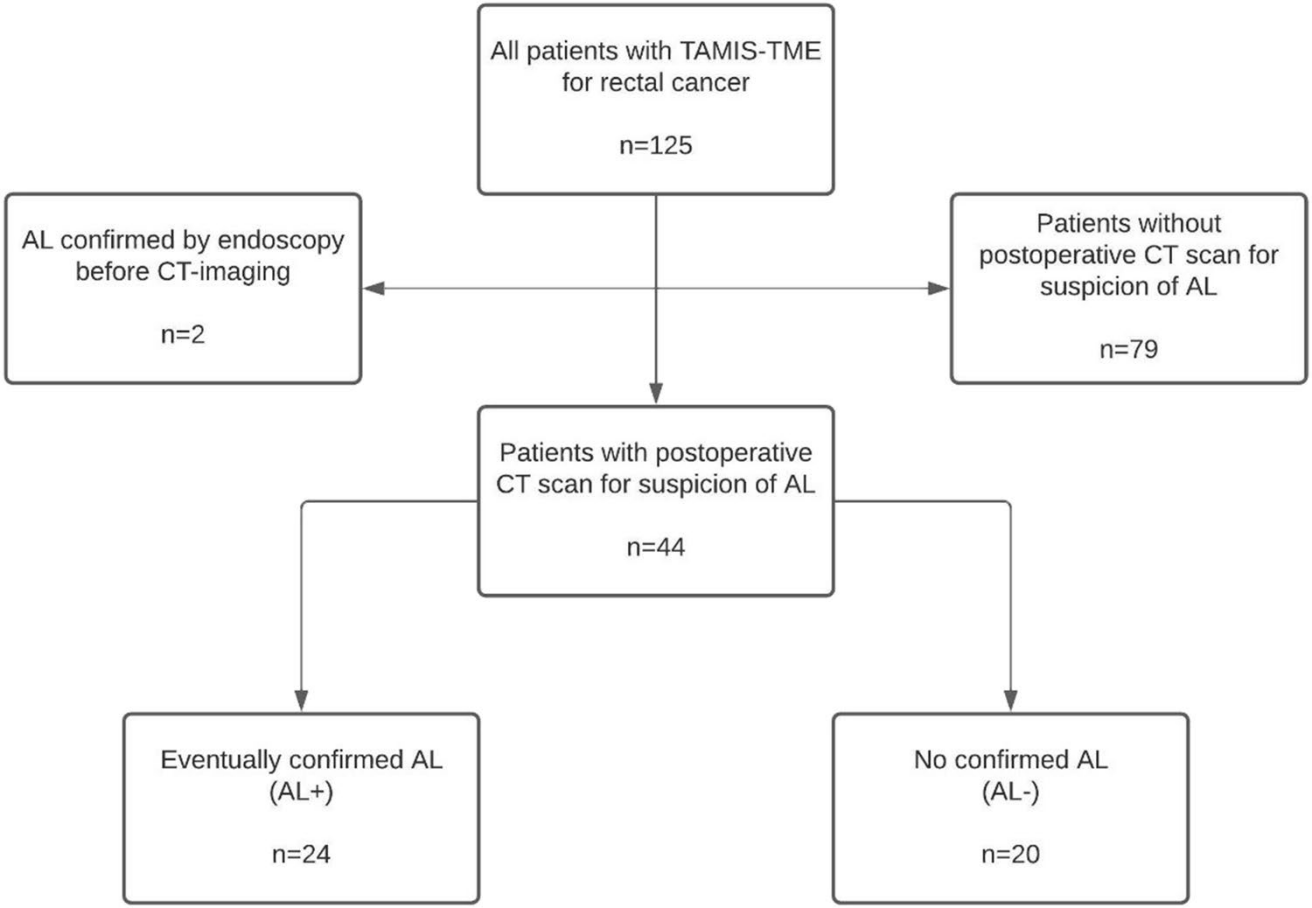
Flowchart of included patients

Out of 125 patients that underwent TaTME for rectal cancer during the study period, 44 patients (35%) underwent a diagnostic CT scan for suspicion of AL (Fig. 1). The mean age was 61 years, mean BMI was 26 kg/m^2^, and 34 (77%) were male. Preoperative radiotherapy was given in 22 patients (50%), and 7 patients had a diverted anastomosis (16%) (Table 1).

**Table 1:** Demographics of 44 patients who underwent CT scan for suspected anastomotic leakage after TME for rectal cancer, stratified for confirmed leakage (AL +) by surgery and/or endoscopy or no leakage (AL −)

	Total (*n* = 44)	AL + (*n* = 24)	AL − (*n* = 20)	*p* value
Mean age in years [SD]	61 ± 9	60 ± 9	61 ± 9	0.859
Gender (male)	34 (77%)	19 (79%)	15 (75%)	0.743
Mean BMI (kg/m2) [SD]	26 ± 4	25 ± 4	26 ± 4	0.835
Smoker	5 (11%)	1 (4%)	4 (20%)	0.099
ASA score				
I	10 (23%)	7 (29%)	3 (15%)	0.319
II	33 (75%)	17 (71%)	16 (80%)	
III + IV	1 (2%)	0	1 (5%)	
Neoadjuvant radiotherapy	22 (50%)	10 (42%)	12 (60%)	0.226
Stoma after LAR	7 (16%)	5 (21%)	2 (10%)	0.328
Preoperative colostomy	1 (2%)	1 (4%)	0	
No stoma	37 (84%)	19 (79%)	18 (90%)	0.328
Anastomotic technique				
Stapled	40 (91%)	20 (83%)	20 (100%)	0.056
Hand-sewn	4 (9%)	4 (17%)	0	
Anastomotic configuration				
Side-to-end	29 (66%)	14 (58%)	15 (75%)	0.895
End-to-end	15 (34%)	10 (42%)	5 (25%)	

*AL* anastomotic leakage, *SD* standard deviation, *BMI* body mass index in kilograms (kg) per square meter (m^2^), *ASA-score* American Society of Anesthesiologists physical status

### CT imaging and radiological features

Reason for performing CT imaging was elevated CRP level in 10 patients, a combination of elevated CRP and clinical signs in 18 patients, and clinical signs of AL in 16 patients. CT imaging was performed after median 148 h (IQR 94–335) from index surgery and after median 5 h (IQR 3–6) from last CRP measurement preceding CT imaging. Of all patients, 40 (91%) received at least rectal contrast (Table 2)*.* No complications of contrast administration were registered.

**Table 2:** Characteristics of CT-imaging for the total cohort of 44 patients with suspected leakage after low anterior resection, and for those with (AL +) or without (AL −) confirmed leakage

	Total (*n* = 44)	AL + (*n* = 24)	AL − (*n* = 20)	*p* value
Contrast				
IV + oral + rectal	17 (39%)	11 (46%)	6 (30%)	0.455
IV + rectal	22 (50%)	12 (50%)	10 (50%)	
IV + oral	3 (7%)	1 (4%)	2 (10%)	
Oral + rectal	1 (2%)	0	1 (5%)	
IV only	1 (2%)	0	1 (5%)	
Radiological feature				
Abscess near anastomosis	2 (5%)	2 (8%)	0	0.186
Abscess not near anastomosis	13 (30%)	8 (33%)	5 (25%)	0.546
Contrast extravasation^*^	18 (45%)	18 (78%)	0	0.000
Fluid around anastomosis	12 (27%)	8 (33%)	4 (20%)	0.323
Free fluid intra-abdominally	14 (32%)	5 (21%)	9 (45%)	0.087
Air around anastomosis^#^	20 (46%)	15 (63%)	5 (25%)	0.013
Free air intra-abdominally	25 (57%)	17 (71%)	8 (40%)	0.040
Extraluminal air^#^	23 (52%)	17 (71%)	6 (30%)	0.007
Fat infiltration	13 (29%)	6 (25%)	7 (35%)	0.469
Presacral collection	12 (28%)	8 (35%)	4 (20%)	0.281
Vaginal fistula	2 (5%)	2 (8%)	0	0.186
Other CT findings				
Paralytic ileus	9 (21%)	5 (21%)	4 (20%)	0.946
Bladder wall thickening	2 (5%)	1 (4%)	1 (5%)	0.895
Pancreatitis	3 (7%)	1 (4%)	2 (10%)	0.445
Sigmoid perforation	1 (2%)	0	1 (5%)	0.268
Air around vaginal top	1 (2%)	0	1 (5%)	0.268
Strangulation ileus	1 (2%)	0	1 (5%)	0.268
Retention bladder	1 (2%)	0	1 (5%)	0.268
Wound infection	1 (2%)	0	1 (5%)	0.268
Perihepatic fluid collection	1 (2%)	0	1 (5%)	0.268

*CT* computed tomography, *AL* anastomotic leakage, *IV* intravenous

^*^Percentage corresponds with total number of patients that received rectal contrast (*n* = 40), of which 23 in the AL + group and 17 in the AL − group

^#^Extraluminal air and air around anastomosis are reported separately based on radiological reports

AL was eventually confirmed in 24 of 44 patients with CT imaging for suspected AL (55%). CT scan was conclusive or highly suspicious of AL in 23 of those 24 cases, resulting in a yield of 23/44 (52%). The only false-negative finding (sensitivity 96%) was in a patient with a diverting ileostomy who had an initial negative CT scan for AL on POD 3. During routine follow-up at 2 weeks, a leak was found by endoscopy and subsequently treated. In another patient with an eventually confirmed AL, the first CT scan could not be adequately assessed due to artifacts caused by total hip prostheses. This patient underwent a second CT scan the next day, which was conclusive for AL. In a third patient, explorative laparoscopy for suspected AL revealed peritonitis without a defect of the anastomosis and abdominal washout with formation of an ileostomy was performed. During repeat endoscopy 4 days later, an anastomotic defect was seen and endosponge treatment started.

In the 20 patients without confirmed AL, the radiology reports indicated no (*n* = 16) or minor (*n* = 4) suspicion for AL (specificity of 100%). Based on CT findings in the AL − group, no endoscopies or surgical explorations with negative findings were performed. There were two patients with eventually confirmed AL who did not initially undergo CT imaging, because diagnosis of AL was confirmed by endoscopy before CT imaging was performed. These two patients were not included in the present analysis.

Four radiological features were significantly more often seen in the AL + group: contrast extravasation in 78% vs 0% (*p* = 0.000), air around the anastomosis in 63% vs 25% (*p* = 0.013), intra-abdominal free air in 71% vs 40% (*p* = 0.040) and extraluminal air in 71% vs 30% (*p* = 0.007). A vaginal fistula was seen in two patients with confirmed AL. The presence of radiological features stratified for confirmed diagnosis of AL is summarized in Table 2.

### Postoperative vital and clinical parameters at time of CT imaging

The presence of vital and other clinical parameters at the time of CT imaging are shown in Table 3. Most of the parameters were not discriminative for AL, except for need for oxygen (17% vs 0%, *p* = 0.05) and abnormal temperature (48% vs. 15%, *p* = 0.022).

**Table 3:** Vital and clinical parameters on the day of the first postoperative CT scan for suspected anastomotic leakage after low anterior resection, displayed for the total cohort and depending on whether or not the leakage was confirmed by surgery and/or endoscopy

	Total (*n* = 43/44)	AL + (*n* = 23/24)^*^	AL − (*n* = 20)	*p* value
Vital parameters				
Hypotension, (syst. BP < 100 mmHg)	6 (14%)	3 (13%)	3 (15%)	0.853
Tachycardia, (> 100BPM)	13 (30%)	9 (39%)	4 (20%)	0.173
Abnormal temperature^1^	14 (33%)	11 (48%)	3 (15%)	0.022
Fever (*T* > 38 °C)	10 (23%)	8 (35%)	2 (10%)	0.055
Hypothermia (*T* < 36 °C)	4 (9%)	3 (13%)	1 (5%)	0.365
Tachypnea (resp. rate > 20/min)	6 (14%)	4 (17%)	2 (10%)	0.485
Clinical parameters				
Need for oxygen	4 (9%)	4 (17%)	0	0.050
Pelvic pain	35 (81%)	20 (87%)	15 (75%)	0.315
Nausea	27 (63%)	15 (65%)	12 (60%)	0.724
Vomiting	22 (51%)	14 (61%)	8 (40%)	0.172
Nasogastric tube	15 (35%)	7 (30%)	8 (40%)	0.512
Abdominal distention	12 (28%)	5 (22%)	7 (35%)	0.334

^*^Vital and clinical parameters of one patient with AL could not be retrieved

*AL* anastomotic leakage, *Syst. BP* systolic blood pressure, *mmHg* millimeters of mercury, *BPM* beats per minute, *T* Temperature, *min* minutes, *resp.* respiratory

^1^Disturbances in temperature defined by either hypo- (*T* < 36 °C) or hyperthermia (*T* > 38 °C)

### Timing of CT scan and corresponding CRP-levels

CT imaging was performed on day 2 after a CRP of 336 mg/L, and on day 3 in 6 patients after a mean CRP of 300 mg/L, which was the only CRP measurement in 3 patients and CRP was measured more than once in the other 3 patients. Ten patients had a CT scan on day 4 after a mean CRP of 283 mg/L, which was the only or repeated CRP measurement in 4 and 6 patients, respectively. A total of 4 CT scans were performed on day 5 after repeated CRP measurement with a mean of 189 mg/L of the CRPs preceding CT imaging. The remaining 23 patients underwent CT imaging for suspected AL later on, and CRP was not measured within 24 h from imaging in 7 of those patients. All CRPs preceding imaging on day 3, 4, or 5 were above the predefined cutoff levels for suspected AL. The proportions of patients with confirmed AL for the different postoperative days of CT imaging were 0/1 (0%) on day 2, 6/6 (100%) on day 3, 7/10 (70%) on day 4, 2/4 (50%) on day 5, and 9/23 (39%) beyond day 5. Table 4 summarizes these data with stratification between confirmed AL or not.

**Table 4:** Timing of CT imaging with corresponding CRP levels and elevated CRP according to predetermined cut-off values for each postoperative day, with subgroup analysis whether or not anastomotic leakage was eventually confirmed

	Total	AL **+ **	AL −	*p* value
CT on day 2	1**/**44	0**/**24	1**/**20	
Mean CRP	336 ± NA	NA	336 ± NA	NA
CT on day 3	6**/**44	6**/**24	0**/**20	
Mean CRP*	300 ± 72	300 ± 72	NA	NA
CRP > 172	6 (100%)	6 (100%)	0	NA
CT on day 4	10**/**44	7**/**24	3**/**20	
Mean CRP*	283 ± 76	293 ± 62	260 ± 115	0.554
CRP > 124	10 (100%)	7 (100%)	3 (100%)	NA
CT on day 5	4**/**44	2**/**24	2**/**20	
Mean CRP*	189 ± 44	193 ± 59	185 ± 47	0.897
CRP > 144	4 (100%)	2 (100%)	2 (100%)	NA
CT beyond day 5	23**/**44	9**/**24	14**/**20	
CRP measured preceding CT	16/44	7/24	9/20	
Mean CRP*	83 ± 61	106 ± 50	66 ± 67	0.209

*AL* Anastomotic leakage, *CRP* C-reactive protein, * = last CRP preceding CT scan in case of multiple measurements

Cutoff values for CRP to predict AL were previously calculated in a review by Singh et al.: CRP > 172 on day 3, CRP > 124 on day 4, and CRP > 144 on day 5

### Reintervention for AL

Reintervention for AL consisted of conservative treatment with antibiotics in 2 patients (8%). Ten patients (42%) received a diverting ileostomy and started EVT, 4 patients (17%) underwent a diverting ileostomy with EVT and suturing of the defect, 5 patients (21%) started with EVT alone, 2 patients (8%) underwent a redo-procedure with ileostomy and 1 patient (2%) underwent an intersphincteric resection of the anastomosis with end-colostomy. No mortality due to AL occurred.

### Timing of CRP-measurement, imaging, and reinterventions

**Fig. 2:**
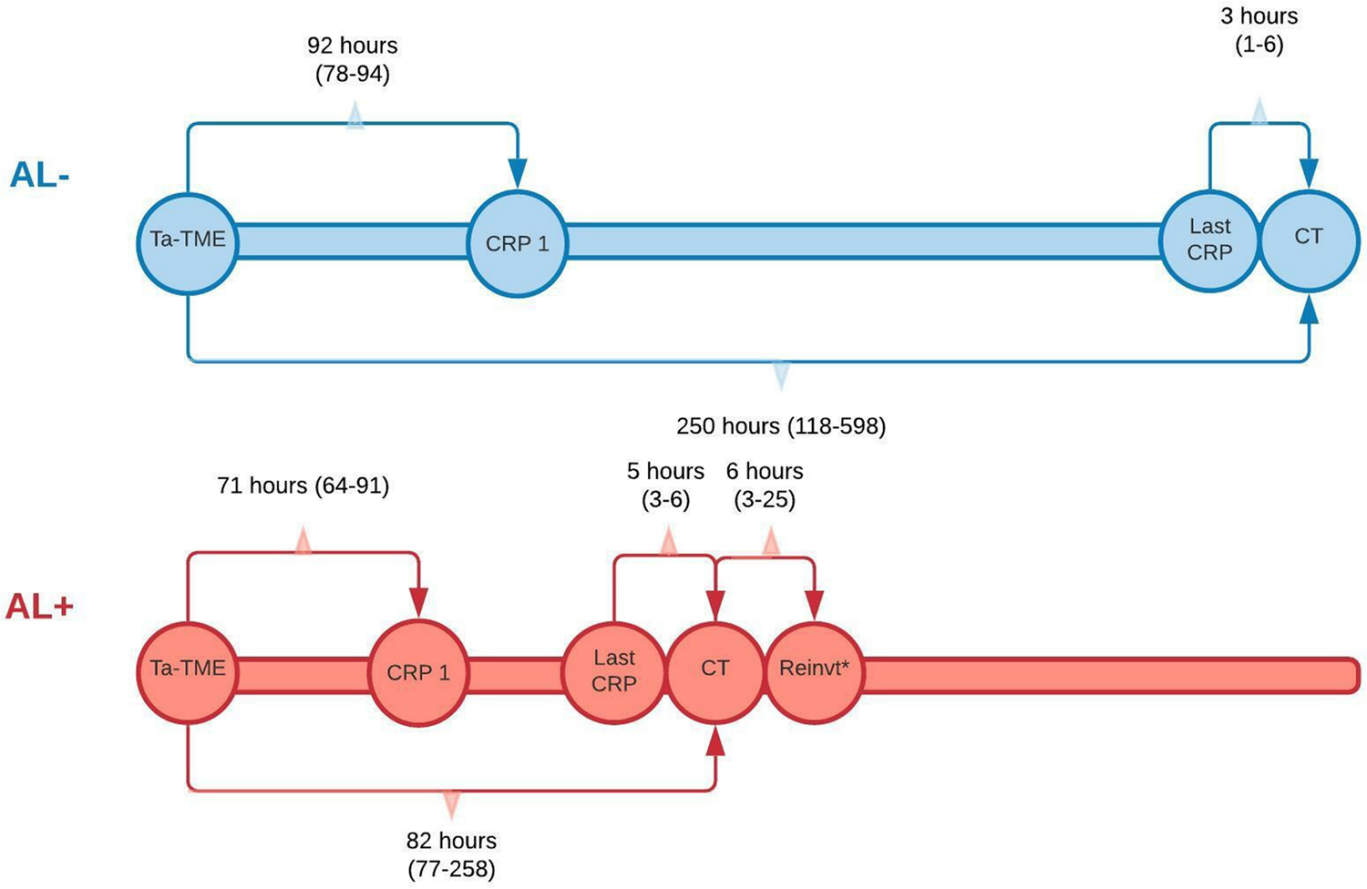
Median time interval in hours between index surgery, CRP measurements, CT imaging, and subsequent reintervention

Median time interval between index surgery and initial CRP measurement was 71 h (64–91) for patients in the AL + group versus 92 h (78–94) in the AL − group (*p* = 0.009). Time interval between index surgery and the first postoperative CT scan in the AL + group was 82 h (77–258) compared to 250 h (118–598) in the AL − group (*p* = 0.020). Time between last CRP and CT imaging in the AL + group was 5 h (3–6) versus 3 h (1–6) in the AL − group (*p* = 0.413). Time from CT imaging to first reintervention in the AL + group was 6 h (3–25). See also Table 5 and Fig. 2.

**Table 5:** Timing of CRP-measurement, imaging, and reinterventions

	Total cohort			AL** + **			AL** − **			
	*n*	Median	IQR	*n*	Median	IQR	*n*	Median	IQR	*p* value
TME — initial CRP, (hours)	42	87	69–94	22	71	64–91	20	92	78–94	0.009
TME — first CT imaging, (hours)	44	148	94–335	24	82	77–258	20	250	118–598	0.020
Initial CRP — last CRP^**1**^, (hours)	35	25	12–144	22	22	6–124	13	68	18–229	0.123
CRP preceding CT^**1**^ — CT, (hours)	35	5	3–6	22	5	3–6	13	3	1–6	0.413
CT — first reintervention, (hours)	24	6	3–25	24	6	3–25	NA	NA	NA	NA

*AL* Anastomotic leakage, *TME* total mesorectal excision, *CRP* C-reactive protein, *CT* computed tomography, *IQR* interquartile range

^1^Last CRP before CT imaging (in case of multiple CRP measurements), only included for patients with serum CRP levels that were measured within 24 h of the CT scan

## Discussion

In this retrospective cohort study, the added value of CRP-guided CT imaging was evaluated for the diagnosis and subsequent management of AL after TME for rectal cancer in a cohort with highly selective diversion. CT imaging was performed in 35% of the initial cohort, which was based on CRP levels above predefined cutoff values on days 3–5 in 45% of those patients. CT imaging in the remaining patients was performed on other postoperative days for different reasons. The overall yield of CT imaging was 52%, with a sensitivity of 96% and a specificity of 100%. CT imaging was performed after median 148 h from TME and 5 h from last CRP. The yield of CT imaging seemed to decrease with increasing interval from TME. Subsequent first reintervention for AL after CT imaging followed after a median of 6 h, and no endoscopic or surgical interventions with negative findings were performed after a negative CT scan, indicating the added valued of CT in timely and tailored re-intervention using this institutional protocol.

Compared to literature, the present study reveals a relatively high rate of positive CT scans (55%) and low rate of false-negative (2%) and false-positive (0%) findings [11, 16]. A recent study including patients that underwent CT imaging for AL-suspicion after colorectal surgery found 24.8% of scans positive for AL with a 32% false-negative and 7% false-positive rate [17]. A possible explanation for the high yield of CT imaging is the use of a postoperative protocol with routine CRP measurement, which increases the a priori likelihood of AL in the tested population. This protocol might also increase diagnostic accuracy, besides the close collaboration and joined effort by clinicians and radiologists in our unit to interpret the images and to take all available clinical, laboratory and radiological signs of AL into account for a definitive diagnosis.

It is also important to emphasize the specific setting of this study with highly selective diversion. The diagnosis of AL is often more clear in the absence of a diverting stoma and this might have also contributed to the high diagnostic accuracy. Especially in those patients with early clinical signs of AL, there is a high yield of CT imaging: 15 confirmed ALs out of 20 CT scans performed on postoperative days 3–5 (75%). Diagnosing AL might be more difficult in case of routine diversion because of masked clinical signs of AL or even asymptomatic leaks.

Timing of CT scanning is essential for adequate detection of AL, because it might take some time before an abscess cavity behind the anastomosis becomes visible. In defunctioned cohorts, it has been suggested that CT-imaging should be performed at least 7 days postoperatively [16, 18]. The present study suggests that CRP measurements can facilitate timely CT imaging with a high yield early on in the postoperative period, although this cannot be extrapolated to studies with routine fecal diversion. Rectal contrast is another valuable element of CT imaging for this purpose, although a fluid collection in contact with the anastomosis not containing contrast is also highly suspicious. A retrospective cohort study with 108 patients that received a CT scan within 16 days after colorectal surgery found that fluid near the anastomosis, air near the anastomosis, intra-abdominal air, and contrast leakage to be highly associated with AL [10]. Another study showed similar results; of the patients with an AL, 32/33 (97%) had contrast extravasation on their CT, and 97/114 (85%) had a perianastomotic fluid collection [11]. We found a presacral collection in 20% of patients without confirmed leakage, which reveals that this should be interpreted with caution. Size of the collection and increase in size over time might be more specific for AL [19]. There are also pitfalls related to the rectal contrast. At an early stage, the defect could be too small to permit extraluminal flow of contrast. By overinflating the balloon, a (small) defect in a low anastomosis could have been sealed during imaging. Finally, inadequate contrast administration might lower the sensitivity in detecting AL [17]. Subsequently false negative imaging may lead to delay in reintervention and increased mortality [20].

Singh et al. calculated the predictive value of serum CRP levels on postoperative days 3, 4, and 5 [9]. In this review, patient groups were heterogenous, both segmental colon and rectal resections were included, and diversion rates were unclear. Diversion is less common after segmental colon resections, which probably increases the validity of these data for our cohort of highly selective diversion. All confirmed leaks had a CRP level above the published cutoff levels by Singh et al. However, once CT imaging has been performed based on these CRP cutoff values, CRP is no longer predictive for AL given the similar CRP levels in those patients without confirmed AL. Delay in elevation of CRP is possible and may be normal due to surgical stress [21, 22]. Repeat measurements might be necessary as some patients in this cohort had normal CRP levels on day 3, but showed increased CRP levels on day 4 or day 5.

All patients in this study cohort were operated on in an academic teaching hospital with a proactive treatment approach to AL. Endoscopy was often part of subsequent interventions following CT imaging. Endoscopy is able to confirm suspected AL based on CT, but this requires specific expertise. A small area of granulation tissue without visible defect might hide the leak. If there is presacral collection on CT, probing of such an area with a forceps or guide wire should then be performed to prove the diagnosis of AL. Furthermore, endoscopic inspection of the anastomosis can be valuable to determine the exact size of the defect and whether there is retraction or ischemia of the afferent loop. More research may be needed to investigate the accuracy and additional value of endoscopy versus CT in detecting AL as a single or combined diagnostic modality.

Our AL rate of 19% (24/125) seems higher than often reported. Clinical AL rates might be lower in case of routine diversion in combination with a relatively limited follow-up, mostly 30 to 90 days postoperatively. If patients are diverted, diagnosis of AL might occur only following closure of the diverting stoma after several months. In addition, asymptomatic leaks in diverted patients are often not reported. In a Dutch national cross-sectional study, the initially reported 30-day AL rate of 8.2% in the national audit appeared to be actually 13.4% when reviewing patient files in detail, and at 1 year this increased to 20% [4]. We also investigated our own transition from standard to selective diversion and found similar AL rates, similar end-colostomy rates, but much higher long-term ileostomy rates after routine diversion, because many temporary ileostomies are never closed unintentionally [14, 23].

Limitations of this study are the relatively small study population and retrospective design. Initial CRP levels were not measured on the same day for all patients due to changes in protocol, as was stated before. In the review by Sing et al., as referred to in the methods [9], the negative predictive values on days 3 and 4 were similar and should not influence the results in a significant way. The original radiological reports were used in the analysis, without interpretation of the features. Experience of the radiologist and explicit reporting of relevant features could have influenced results. However, we aimed to determine the value of CT based on routine daily practice, for which reason we decided not to revise the images by expert radiologists or second readers.

In conclusion, this study showed a high yield of CT imaging in an academic center with a policy of highly selective fecal diversion after TME for rectal cancer and a pro-active leakage management. CT imaging can be performed in an early postoperative setting based on elevated CRP levels above published cutoff values for postoperative days 3–5, together with other clinical signs of AL. This allows for timely and tailored subsequent reintervention for AL within a few hours, and prevents overtreatment with negative explorative interventions at the same time.

## Supplementary Information

Below is the link to the electronic supplementary material.

Supplementary file1 (DOCX 19 KB)
