# Supplementary material for: Usefulness of CT scan as part of an institutional protocol for proactive leakage management after low anterior resection for rectal cancer
Source: Langenbecks Arch Surg. 2022 Aug 25;407(8):3567–75. doi: 10.1007/s00423-022-02652-z (PMC9722798; doi:10.1007/s00423-022-02652-z)
Supplement: Supplementary file 1 — Supplementary file1 (DOCX 19 KB) [file 423_2022_2652_MOESM1_ESM.docx]

Supplementary table 1: Tumor Characteristics

|  | **Total**  **(n=44)** | **AL +**  **(n=24)** | **AL -**  **(n=20)** | **p-value** |
| --- | --- | --- | --- | --- |
| **T-stage** |  |  |  |  |
| T1 | 3 (7%) | 1 (4%) | 2 (10%) | 0.135 |
| T2 | 13 (30%) | 7 (29%) | 6 (30%) |  |
| T3a | 10 (22%) | 5 (21%) | 5 (25%) |  |
| T3b | 7 (16%) | 7 (29%) | 0 |  |
| T3c | 7 (16%) | 2 (8%) | 5 (25%) |  |
| T4 | 4 (9%) | 2 (8%) | 2 (10%) |  |
| **N-stage** |  |  |  |  |
| N0 | 21 (48%) | 14 (58%) | 7 (35%) | 0.132 |
| N1 + N2 | 23 (52%) | 10 (42%) | 13 (65%) |  |
| **M- Stage** |  |  |  |  |
| Lung | 2 (5%) | 1 (4%) | 1 (5%) | 0.447 |
| Liver | 4 (9%) | 3 (13%) | 1 (5%) |  |
| Other | 2 (5%) | 2 (8%) | 0 |  |

*TNM-stage* Tumor Nodus Metastasis stage (as preoperatively determined by MRI-scanning), *MRI* magnetic resonance imaging
